# Further dissection of Gardnerella vaginalis: description of Gardnerella lacydonensis sp. nov. (formerly genomic species 2), Gardnerella bretellae sp. nov. (formerly genomic species 9), Gardnerella massiliensis sp. nov. (formerly genomic species 14) and Gardnerella phocaeensis sp. nov.

**DOI:** 10.1099/ijsem.0.007028

**Published:** 2026-03-04

**Authors:** Priscille Allini Ntiguemassa, Mamadou Beye, Nicolas Orain, Sara Bellali, Stephane Alibar, Claudia Andrieu, Alissa Hammoud, Grégory Dubourg, Oleg Mediannikov, Anthony Levasseur, Florence Fenollar

**Affiliations:** 1 Aix Marseille Univ, AP-HM, SSA, RITMES, Marseille, France; 2 Institut Hospitalo-Universitaire Méditerranée Infection, Marseille, France; 3 AP-HM, Marseille, France; 4 IRD, Marseille, France; 5 Aix Marseille Univ, IRD, AP-HM, MEPHI, Marseille, France

**Keywords:** *Gardnerella bretellae *sp. nov., *Gardnerella lacydonensis* sp. nov., *Gardnerella massiliensis *sp. nov., *Gardnerella phocaeensis *sp. nov., *Gardnerella vaginalis*, whole-genome sequencing

## Abstract

Four bacterial strains – Marseille-Q9181^T^, Marseille-QA0894^T^, Marseille-Q9179^T^ and Marseille-Q2328^T^ – were isolated, the first three from vaginal samples and the last from a blood sample. MALDI-TOF MS analysis failed to assign these isolates to any known species within the genus *Gardnerella*. Consequently, phenotypic and taxonogenomic characterizations were conducted. All four strains were Gram-negative, non-motile and non-spore-forming coccobacilli. Strains Marseille-Q9181^T^, Marseille-Q2328^T^ and Marseille-Q9179^T^ were facultative anaerobes with microaerophilic capabilities, whereas Marseille-QA0894^T^ was strictly anaerobic and microaerophilic. The dominant fatty acids (>10%) identified in strains Marseille-Q9181^T^, Marseille-QA0894^T^ and Marseille-Q2328^T^ were C_16 : 0_, C_18 : 1_ ω9c and C_18 : 0_. In strain Marseille-Q9179^T^, C_18 : 0_ was also among the most abundant fatty acids, along with the aforementioned three. Digital DNA–DNA hybridization (dDDH) values for strains Marseille-Q9181^T^, Marseille-QA0894^T^ and Marseille-Q2328^T^ against *Gardnerella vaginalis* 1400E (genomic species 2), *G. vaginalis* 6119V5 (genomic species 9) and *G. vaginalis* NR010 (genomic species 14) were 72.1%, 72.4% and 75.5%, respectively. The corresponding OrthoANI values were 96.99%, 97.16% and 97.66%, all above the species demarcation threshold. In contrast, strain Marseille-Q9179^T^ exhibited both dDDH and OrthoANI values below the accepted thresholds (70% and 95–96%, respectively), suggesting that it represents a novel genomic lineage. Based on comprehensive phenotypic and genomic analyses, we propose that these strains represent four new species within the genus *Gardnerella*, for which the following names are proposed: *Gardnerella lacydonensis* sp. nov. (type strain: Marseille-Q9181^T^=CSUR Q9181^T^=CECT 31121^T^), *Gardnerella bretellae* sp. nov. (type strain: Marseille-QA0894^T^=CSUR QA0894^T^=CECT 31122^T^), *Gardnerella massiliensis* sp. nov. (type strain: Marseille-Q2328^T^=CSUR Q2328^T^=CECT 30239^T^) and *Gardnerella phocaeensis* sp. nov. (type strain: Marseille-Q9179^T^=CSUR Q9179^T^=CECT 31120^T^).

## Introduction

*Gardnerella vaginalis* is a facultative anaerobic bacterium associated with bacterial vaginosis [1,2]. Different classification systems for *G. vaginalis* (biotyping, genotyping and sub-grouping) have been previously proposed [3,6]. However, some *Gardnerella* isolates have never been identified using clade-specific qPCR assays [4,7,9]. Additionally, analysis of the V4 hypervariable region of the 16S rRNA gene revealed that for over 98.5% of *Gardnerella* species, the V4 region was identical [10]. In order to resolve their taxonomic position, 81 genomes of *G. vaginalis* were analysed in 2019. Based on an average nucleotide identity of 96% and DNA–DNA hybridization of 70% or higher, strains of *G. vaginalis* were classified into several genomic species, with description of six species: *G. vaginalis sensu stricto*, and five ex-genomic species as *Gardnerella leopoldii*, *Gardnerella piotii*, *Gardnerella swidsinskii*, *Gardnerella pickettii* and *Gardnerella greenwoodii* [10,11]. Other genomic studies, using a larger number of genomes, have revealed the presence of additional genotypes [12], including fourteenth genomic species (*Gardnerella* NR010 genomic species 14), with an average nucleotide identity value lower than or equal to 96% compared to other genomes, that is compatible to a new species [13,14]. Despite the number of molecular and genomic differences [7,15] among genotypes, some have never been officially described as new species, notably genomic species 2, 7, 9, 10, 11, 12, 13 and 14. Here, we demonstrate genetic and biological differences and describe four species of the genus *Gardnerella*: *Gardnerella lacydonensis*, *Gardnerella bretellae* and *Gardnerella massiliensis*, known as genomic species 2, genomic species 9 and genomic species 14, respectively, as well as *Gardnerella phocaeensis*, a new potential species, not attributed previously to any genomic species. We used phenotypic, taxonomic and genomic approaches to characterize these species.

## Methods

### Ethical considerations and sampling

The four strains were isolated from four specimens (three vaginal swabs and one blood culture) taken during patient care and sent for diagnostic purposes to the clinical microbiology laboratory at our university hospital (AP-HM, at the Institut Hospitalo-Universitaire Méditerranée Infection, Marseille, France). As permitted by French law (Article L.1211-2 of the Public Health Code), patients have been informed of the possible re-use of their samples and personal data collected during treatment for research purposes. They had the option of objecting by notifying the AP-HM Data Protection Officer, but none of the four patients indicated any objections.

### Bacterial colony identification

Bacterial colonies from vaginal swabs and blood samples were isolated on Columbia agar enriched with 5% sheep blood (bioMérieux, Marcy-l'Etoile, France) in an anaerobic atmosphere using a GasPak^™^ EZ Anaerobe gas generating system (Becton Dickinson, Franklin Lakes, NJ, USA) for 48 h. Prior to this, vaginal samples were incubated at 5% CO_2_ for 48 h at 37 °C on three culture media: PolyViteX chocolate agar, PolyViteX VCAT3 chocolate agar and Columbia CNA agar, which was enriched with 5% sheep blood (bioMérieux), whereas the blood sample was incubated in an anaerobic blood culture bottle for 28 h. Subsequently, all bacterial colonies were identified by MALDI-TOF MS using the Microflex LT tool instrument (Bruker Daltonics, Bremen, Germany). A score of >2 indicated correct species identification, while a score of ≥1.7 but <2 indicated correct genus identification to identify colonies of the genus *Gardnerella* [16,17].

### Phenotypic characteristics

#### Growth characteristics

Gram staining of cell was performed using a Color Gram 2 kit (bioMérieux). The staining was evaluated under a Motic Panthera C2 trinocular microscope with a 100X oil immersion lens. The images were produced using Mosaic software version 3.0.7. Cell morphology of the four strains was evaluated by scanning electron microscopy (SEM) using an SU5000 microscope (Hitachi High-Tech Corporation, Tokyo, Japan) [18]. Sporulation and motility were tested as previously described [18]. Moreover, optimal growth conditions were determined on Columbia agar enriched with 5% sheep blood (bioMérieux) for 72 h at different growth temperatures (28, 37, 45 and 56 °C) and different atmospheres: aerobic, anaerobic using the GasPak^™^ EZ Anaerobe gas generation system (Becton Dickinson) and microaerophilic using the GasPak^™^ Campy pocket system (Becton Dickinson). Salt tolerance and ability to grow under different pH conditions were assessed after 24, 48 and 72 h of incubation at 37 °C under anaerobic conditions, using 37 g brain heart infusion broth (Becton Dickinson) supplemented with 5 g yeast extract, 1 g starch, 1 g peptone and 21 g Columbia agar (Thermo Fisher Scientific, Waltham, MA, USA) for 1 l. NaCl was added to obtain salt concentrations of 5%, 7%, 10%, 15%, 20%, 25% and 30%. NaOH or HCl was adjusted to 4.5, 5.5, 6.5, 7.5 and 8.5 to achieve the pH values tested.

#### Biochemical tests

Catalase and oxidase activity were assayed using a hydrogen peroxide solution (bioMérieux) and oxidase discs (Becton Dickinson), respectively, as previously described [19]. Carbohydrate metabolism and enzymatic activities of all strains were analysed using API^®^ ZYM, API^®^ 50 CH and API^®^ 20 A strips (bioMérieux). Cellular fatty acid methyl esters were analysed by GC/MS as already reported [20].

#### Antibiotic susceptibility

Amoxicillin, amoxicillin/clavulanic acid, ticarcillin/clavulanic acid, ceftriaxone, imipenem, vancomycin, colistin, tobramycin, azithromycin, ciprofloxacin, clindamycin, doxycycline and metronidazole were tested for antibiotic susceptibility using E-test strips (bioMérieux). Mueller–Hinton agar supplemented with 5% sheep blood (bioMérieux) was used to test antibiotic susceptibility. The manufacturer’s instructions were followed to read the MIC, which was then interpreted in accordance with the guidelines of the European Committee on Antimicrobial Susceptibility (https://www.eucast.org/) consulted in January 2025 and the CLSI M100 guidelines. When breakpoint values were available, antibiotic susceptibility was interpreted according to the following specific categories: susceptible (S), susceptible, increased exposure (I) and resistant (R). In the absence of breakpoint values and when MICs were high, as for colistin and tobramycin, strains were considered resistant. In other cases (azithromycin, ciprofloxacin and doxycycline), MICs could not be interpreted due to the absence of breakpoint values.

### Whole-genome sequencing and bioinformatics analysis

Genomic DNA from the four strains was extracted with the EZ1 instrument (Qiagen, Hilden, Germany) using the EZ1 DNA Tissue kit (Qiagen) and was sequenced on the MiSeq sequencer (Illumina Inc, San Diego, CA, USA) using a Nextera XT Paired End sample preparation kit (Illumina) [21]. Assembled reads were obtained using SPAdes 3.11.1 software [22]. Scaffolds of less than 800 bp and depth values of less than 25% of the median depth were excluded during assembly, and CheckM was used to evaluate the quality of genome assemblies [23]. Genomic sequences for the four strains were annotated using Prokka version 1.14.6 and Rapid Annotation Using Subsystem Technology (RAST) [24,25]. In addition, we performed a BLASTp analysis against the Clusters of Orthologous Genes (COGs) database [26]. Genome maps were constructed using the CGview/Proksee web server [27]. The genomes of the four *Gardnerella* strains were compared to the closely related *Gardnerella* genomic species described in previous studies [10,14].

The 16S rRNA sequences of the 4 strains analysed and the genomes of 14 *Gardnerella* species (Table S1, available in the online Supplementary Material) were aligned using muscle [28]. mega version 11 software was used to construct the phylogenetic tree with 1,000 bootstrap replications using the neighbour-joining method and the Tamura 3-parameter method [29,32]. According to digital DNA–DNA hybridization (dDDH) and the average nucleotide identity (OrthoANI), the degree of pairwise similarity between the genomes was compared with the 14 genomic species of the genus *Gardnerella* [33,34]. The dDDH was calculated by downloading the genome sequences from the Genome-to-Genome Distance Calculator 2.1 (GGDC) web server (http://ggdc.dsmz.de/) consulted in January 2025 [35]. OrthoANI was analysed using OAT version 0.93.1 software, and a heat map was generated [36].

CRISPR loci and CRISPR-Cas clusters with an evidence level of 3 or 4 were analysed using regularly spaced short palindromic repeats (CRISPR). Prophage sequences were identified using the PHASTEST [37]. The insertion sequences and replicon of the plasmids were determined using the ISfinder and PlasmidFinder [38,39]. BAGEL software was used to predict bacteriocins [40], while secondary metabolites were estimated using antiSMASH 5.0 [41]. Genes coding for vaginolysin and sialidase were identified via BLASTp with an identity threshold of 40% and a minimum length of 30% against two databases created with protein sequences of different types of vaginolysin (Type 1A, Type 1B, Type 1C, Type 2 and Type 3) [42] and different sequences of sialidase (*nanH1*, *nanH2* and *nanH3*) [43]. The vaginolysin protein sequences were aligned using muscle [28]. Structural prediction of the candidate proteins vaginolysin and sialidase was performed using PHYRE 2.2 [44].

## Results

### Strain identification by MALDI-TOF MS Biotyper

Strains Marseille-Q9181^T^, Marseille-QA0894^T^ and Marseille-Q9179^T^ were isolated from vaginal samples, while strain Marseille-Q2328^T^ was isolated from a blood sample. MALDI-TOF MS analysis failed to identify the *Gardnerella* strains to the species level. Strains Marseille-Q9181^T^ and Marseille-Q2328^T^ were identified as *G. vaginalis* with scores of 2.01–2.1 and 2.29–2.23, respectively. Strains Marseille-QA0894^T^ and Marseille-Q9179^T^ were identified as *G. leopoldii_swidsinskii* with scores of 2.18–2.33 and 2.18–2.46, respectively.

### Phenotypic characterization

The phenotypic characteristics of the four *Gardnerella* strains were compared with those of other described *Gardnerella* species with validly published names, namely *G. vaginalis*, *G. leopoldii*, *G. piotii*, *G. swidsinskii* [10], *G. pickettii* and *G. greenwoodii* [11].

### Bacterial growth and morphological observations

Growth of the four strains was observed on Columbia agar enriched with 5% sheep blood (bioMérieux), after 48 h of incubation at an optimum temperature of 37 °C in microaerophilic and anaerobic atmospheres. Aerobic growth was only observed for strains Marseille-Q2328^T^ and Marseille-Q9179^T^. Strains Marseille-Q2328^T^ and Marseille-QA0894^T^ grew at a pH ranging from 6.5 to 8.5 (optimum 7.5), while strains Marseille-Q9181^T^ and Marseille-Q9179^T^ grew at a pH ranging from 7.5 to 8.5 (optimum 7.5). Growth was observed at 0.5% NaCl for all four isolates. Regarding morphological description, strain Marseille-Q9181^T^ exhibited thick, whitish bacterial colonies with regular edges. Strain Marseille-QA0894^T^ featured domed colonies with irregular edges and a whitish colour. Strain Marseille-Q2328^T^ exhibited small, circular, flat and transparent colonies. Strain Marseille-Q9179^T^ showed small, circular, whitish-greyish colonies with regular margins.

### Gram staining and SEM

**Fig. 1.**
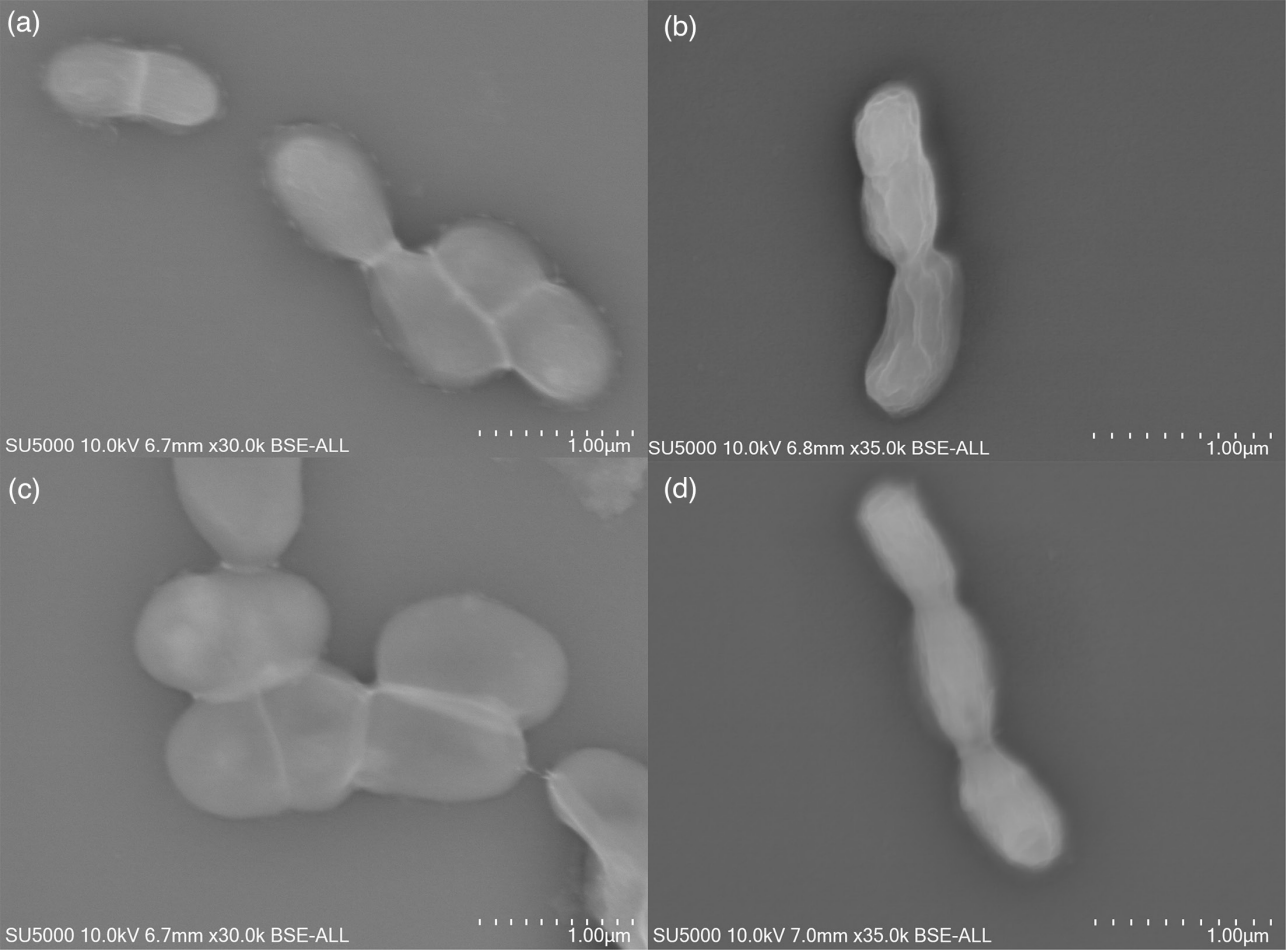
SEM micrographs of (**a**) *G. lacydonensis* Marseille-Q9181^T^, (**b**) *G. bretellae* Marseille-QA0894^T^, (**c**) *G. massiliensis* Marseille-Q2328^T^ and (**d**) *G. phocaeensis* Marseille-Q9179^T^. SEM acquisition settings appear on each micrograph as follows: instrument, accelerating voltage, working distance, magnification, detector and scale.

All four strains stained consistently as Gram-negative coccobacilli (Fig. S1), as well as non-spore-forming and non-motile. SEM micrographs revealed that the cells of strain Marseille-Q9181^T^ measured 1.39±0.4 µm in length and 0.86±0.07 µm in width (Fig. 1a); strain Marseille-QA0894^T^ measured 0.73±0.13 µm in length and 0.5±0.06 µm in width (Fig. 1b); strain Marseille-Q2328^T^ measured 3±0.64 µm in length and 1.5±0.09 µm in width (Fig. 1c); and strain Marseille-Q9179^T^ measured 1±0.05 µm in length and 0.54±0.04 µm in width (Fig. 1d). The growth characteristics of strains Marseille-Q9179^T^, Marseille-Q2328^T^, Marseille-QA0894^T^ and Marseille-Q9179^T^, compared with the most closely related species of the genus *Gardnerella*, are listed in Table 1.

**Table 1.** Phenotypic characteristics of strains *G. lacydonensis* Marseille-Q9181^T^, *G. bretellae* Marseille-QA0894^T^, *G. massiliensis* Marseille-Q2328^T^ and *G. phocaeensis* Marseille-Q9179^T^ compared with *G. vaginalis* ATCC 14018^T^, *G. leopoldii* UGent 06.41^T^, *G. swidsinskii* GS 9838-1^T^, *G. piotii* UGent 18.01^T^*, G. greenwoodii* c31Ua_26^T^ and *G. pickettii* c17Ua_112^T^

**Species**	*G. lac* Marseille-Q9181^T^	*G. bret* Marseille-QA0894^T^	*G. mas* Marseille-Q2328^T^	*G. pho* Marseille-Q9179^T^	*G. vag* ATCC 14018**^T^**	*G. leo* UGent 06.41**^T^**	*G. swid* GS 9838–1**^T^**	*G. piot* UGent 18.01**^T^**	*G. green* c31Ua_26**^T^**	*G. pick* c17Ua_112**^T^**
**Sample origin**	Vagina	Vagina	Blood	Vagina	Vagina	Vagina	Vagina	Vagina	Urine	Urine
**CO_2_ requirement**	5%	5%	5%	5%	5%	5%	5%	5%	5%	5%
**Colony aspect**	Thick, whitish with regular edges	Rounded with irregular edges and whitish	Small, circular and flat, very transparent	Small, circular, whitish greyish with regular edges	Smooth surface from white to greyish	Smooth surface from white to greyish	Smooth surface from white to greyish	Smooth surface from white to greyish	Very small, smooth and circular, light grey, almost transparent	Small, circular and opaque white to greyish
**Cell shape**	Coccobacilli	Coccobacilli	Coccobacilli	Coccobacilli	Coccobacilli	Coccobacilli	Coccobacilli	Coccobacilli	Coccobacilli	Coccobacilli
**Cell size**	1.39×0.86 µm	0.73×0.5 µm	3×1.5 µm	1×0.54 µm	0.5×1.5 µm	0.5×1.5 µm	5×1.5 µm	0.5×1.5 µm	0.94 µm	0.79 µm
**Gram straining**	Negative	Negative	Negative	Negative	Negative to Gram-variable	Negative to Gram-variable	Negative to Gram-variable	Negative to Gram-variable	Positive	Negative
**References**	This study	This study	This study	This study	[10]	[10]	[10]	[10]	[11]	[11]

*G. lacydonensis* sp. nov. (*G. lac* sp. nov.),* G. bretellae* sp. nov. (*G. bret* sp. nov.), *G. massiliensis* sp. nov. (*G. mas* sp. nov.),* G. phocaeensis* sp. nov. (*G. pho* sp. nov.), *G. vaginalis* (*G. vag*), *G. leopoldii* (*G. leop*), *G. swidsinskii* (*G. swid*), G. *piotii* (*G. piot*), *G. greenwoodii* (*G. green*) and *G. pickettii* (*G. pick*).

### Biochemical characterization, antibiotic susceptibility and fatty acids

Strains Marseille-Q2328^T^, Marseille-QA0894^T^, Marseille-Q9181^T^ and Marseille-Q9179^T^ were negative for catalase and oxidase. Using the API^®^ 20 A strip, positive reactions were observed for d-glucose, d-mannitol, lactose, d-sucrose, d-maltose, salicin, aesculin, iron citrate, glycerol, d-cellobiose, d-mannose, d-melezitose and d-sorbitol for all four strains. l-Rhamnose and d-trehalose were positive only for strain Marseille-Q9179^T^. Using the API^®^ 50 CH strip, all four strains were positive for starch. Using API^®^ ZYM strips, all four strains were positive for alkaline phosphatase, leucine arylamidase, valine arylamidase, acid phosphatase, naphthol-AS-BI-phosphohydrolase and α-glucosidase. Strain Marseille-Q9179^T^ was positive for esterase (C4), esterase lipase (C8), lipase (C14) and ß-glucuronidase. Strain Marseille-Q9181^T^ was also positive for ß-galactosidase and d-mannosidase. These results are summarized in Table 2.

**Table 2.** Biochemical characteristics of strains *G. lacydonensis* Marseille-Q9181^T^, *G. bretellae* Marseille-QA0894^T^, *G. massiliensis* Marseille-Q2328^T^ and *G. phocaeensis* Marseille-Q9179^T^

**Species**	*G. lacydonensis* Marseille-Q9181^T^	*G. bretellae* Marseille-QA0894^T^	*G. massiliensis* Marseille-Q2328^T^	*G. phocaeensis* Marseille-Q9179^T^
**Activity of:**				
Oxidase	−	−	−	−
Catalase	−	−	−	−
**Fermentation of:**				
d-Glucose	+	+	+	+
Ribose	−	−	−	−
d-Mannitol	+	+	+	+
Lactose	+	+	+	+
d-Sucrose	+	+	+	+
d-Maltose	+	+	+	+
Salicin	+	+	+	+
Aesculin	+	+	+	+
Iron citrate	+	+	+	+
Glycerol	+	+	+	+
d-Cellobiose	+	+	+	+
d-Mannose	+	+	+	+
d-Melezitose	+	+	+	+
d-Sorbitol	+	+	+	+
l-Rhamnose	−	−	−	+
d-Trehalose	−	−	−	+
Starch	+	+	+	+
**Production of:**				
Acid phosphatase	+	+	+	+
Alkaline phosphatase	+	+	+	+
Valine arylamidase	+	+	+	+
Leucine arylamidase	+	+	+	+
Naphthol-AS-BI-phosphohydrolase	+	+	+	+
Esterase (C4)	−	−	−	+
Esterase lipase (C8)	−	−	−	+
Lipase (14)	−	−	−	+
ß-Galactosidase	+	−	−	−
α-Glucosidase	+	+	+	+
ß-Glucosidase	−	−	−	−
ß-Glucuronidase	−	−	−	+
d-Mannosidase	+	−	−	−

+: positive reaction; −: negative reaction.

MICs for the four strains are shown in Table 3. Briefly, strains Marseille-Q9181^T^, Marseille-QA0894^T^, Marseille-Q2328^T^ and Marseille-Q9179^T^ were susceptible to clindamycin but resistant to metronidazole. The fatty acid composition of the four strains is presented in Table 4 and compared with *G. vaginalis* strains [45]. The most abundant fatty acids (representing >20% of the total) for strain Marseille-Q2328^T^ were C_16 : 0_ (43.7%) and C_18 : 1_ ω9c (28.5%). The most prevalent fatty acid for strain Marseille-Q9181^T^ was C_16 : 0_ (50.2%). For strain Marseille-QA0894^T^, the major fatty acids were C_16 : 0_ (39.2%) and C_18 : 1_ ω9c (20%). For strain Marseille-Q9179^T^, the most abundant fatty acid was C_16 : 0_ (40.3%).

**Table 3.** Antibiotic susceptibility strains *G. lacydonensis* Marseille-Q9181^T^, *G. bretellae* Marseille-QA0894^T^, *G. massiliensis* Marseille-Q2328^T^ and *G. phocaeensis* Marseille-Q9179^T^ to 13 antibiotics, interpreted in accordance with EUCAST and CLSI M100 guidelines

Antibiotics	*G. lacydonensis* Marseille-Q9181^T^		*G. bretellae* Marseille-QA0894^T^		*G. massiliensis* Marseille-Q2328^T^		*G. phocaeensis* Marseille-Q9179^T^	
	MIC (µg ml^−1^)	S/R/I	MIC (µg ml^−1^)	S/R/I	MIC (µg ml^−1^)	S/R/I	MIC (µg ml^−1^)	S/R/I
**Amoxicillin**	0.047	S	0.125	S	0.032	S	<0.064	S
**Amoxicillin/clavulanic acid**	<0.016	S	0.094	S	<0.016	S	0.094	S
**Ticarcillin/clavulanic acid**	0.047	S	0.25	S	0.25	S	1	S
**Ceftriaxone**	0.25	S	0.5	S	<0.016	S	0.25	S
**Imipenem**	0.094	S	0.5	S	0.008	S	0.125	S
**Vancomycin**	0.25	S	0.5	S	2	S	0.5	S
**Clindamycin**	<0.016	S	0.25	S	0.047	S	<0.016	S
**Metronidazole**	≥256	R	16	R	≥256	R	≥256	R
**Colistin**	≥256	R	≥256	R	≥256	R	≥256	R
**Tobramycin**	16	R	≥256	R	≥256	R	≥256	R
**Azithromycin**	<0.016	na	0.023	na	0.064	na	0.032	na
**Ciprofloxacin**	0.5	na	0.75	na	1.5	na	2	na
**Doxycycline**	0.25	na	0.5	na	32	na	32	na

I, susceptible increased exposure; MIC, minimal inhibitory concentration; na, not applicable (MIC could not be interpreted due to the lack of breakpoint values); R, resistant; S, susceptible.

**Table 4.** Fatty acid composition (%) of strains *G. lacydonensis* Marseille-Q9181^T^, *G. bretellae* Marseille-QA0894^T^, *G. massiliensis* Marseille-Q2328^T^ and *G. phocaeensis* Marseille-Q9179^T^ compared to *G. vaginalis* strains

Fatty acids	Names	*G. lacydonensis* Marseille-Q9181^T^	*G. bretellae* Marseille-QA0894^T^	*G. massiliensis* Marseille-Q2328^T^	*G. phocaeensis* Marseille-Q9179^T^	*G. vaginalis* (cluster 1, *n*=40)	*G. vaginalis* (cluster 2, *n*=10)
		Mean relative % (a)					
16 : 0	Hexadecanoic acid	50.2±1.4	39.2±0.8	43.7±1.2	40.3±0.1	36.6±2.9	23.5±2.2
18 : 1 n9	9-Octadecenoic acid	15.3±1.1	20±1.2	28.5±0.4	18±1	35.2±2.2	44.3±3.3
18 : 0	Octadecanoic acid	12.2±0.2	15.1±0.9	12.8±0.4	13.9±2	9.2±1.4	3.8±0.5
14 : 0	Tetradecanoic acid	7.7±0.1	7.6±0.9	4.7±0.1	12.3±2.4	6.9±1.5	4.6±0.4
18 : 2 n6	9,12-Octadecadienoic acid	5.4±0.5	10.1±0.1	5.1±0.6	7.7±0.8	4.8±1.3	8±1.5
10 : 0	Decanoic acid	2.3±0.4	1±0.3	tr	1.2±0.1	nd	nd
18 : 1 n7	11-Octadecenoic acid	1.5±0.2	1.1±0.1	tr	tr	2.5±2	4.4±2.3
17 : 0	Heptadecanoic acid	tr	1±0.1	tr	tr	nd	nd
12 : 0	Dodecanoic acid	1.8±0.6	tr	tr	tr	nd	nd
15 : 0	Pentadecanoic acid	1±0.2	tr	1.1±0.1	tr	nd	nd
16 : 1 n7	9-Hexadecenoic acid	nd	tr	tr	tr	3±0.9	6.2±0.5
19 : 0	Nonadecanoic acid	nd	nd	nd	tr	tr	1.1±0.6
18 : 1 n5	13-Octadecenoic acid	nd	tr	nd	tr	nd	nd
17 : 0 anteiso	14-Methyl-hexadecanoic acid	tr	tr	tr	tr	nd	nd
20 : 4 n6	5,8,11,14-Eicosatetraenoic acid	nd	tr	nd	tr	nd	nd
16 : 1 n9	7-Hexadecenoic acid	nd	tr	tr	tr	nd	nd
17 : 1 n7	10-Heptadecenoic acid	nd	tr	tr	tr	nd	nd
15 : 0 anteiso	12-Methyl-tetradecanoic acid	tr	tr	tr	tr	nd	nd
17 : 0 iso	15-Methyl-hexadecanoic acid	nd	tr	tr	tr	nd	nd
16 : 0 iso	14-Methyl-pentadecanoic acid	nd	tr	tr	tr	nd	nd
15 : 0 iso	13-Methyl-tetradecanoic acid	tr	nd	tr	tr	nd	nd
19 : 01	Nonadecenoic acid	nd	nd	nd	tr	nd	nd
08 : 0	Octanoic acid	nd	nd	nd	tr	nd	nd
14 : 0 iso	12-Methyl-tridecanoic acid	nd	nd	tr	tr	nd	nd
References		This study	This study	This study	This study	[45]	[45]

tr, trace amount <1%; a, mean peak area percentage; nd, not determined; n, number of samples tested.

### Genomic characterization

#### Genome properties

**Fig. 2.**
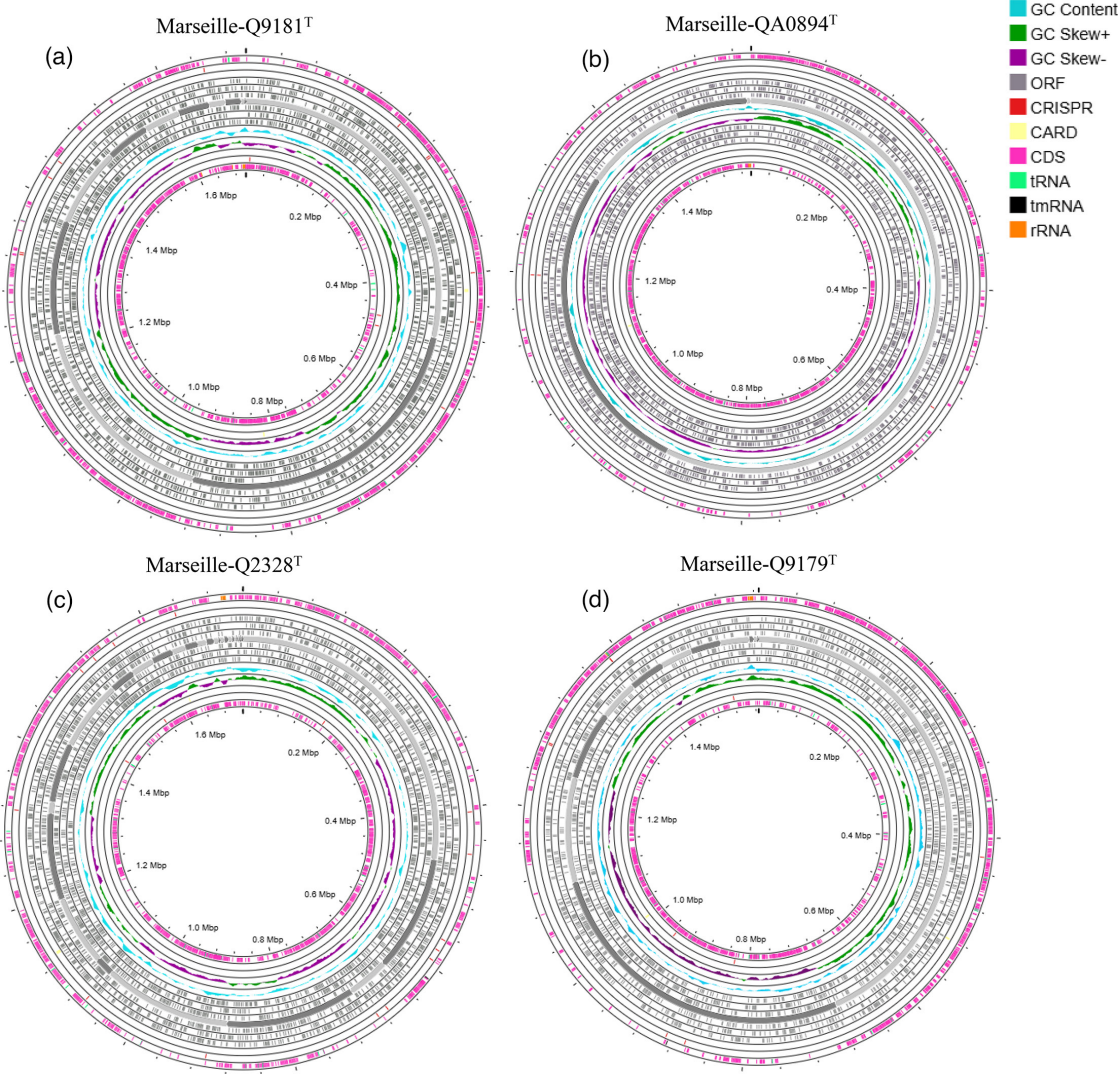
Circular genome map of strains (**a**) *G. lacydonensis* Marseille-Q9181^T^, (**b**) *G. bretellae* Marseille-QA0894^T^, (**c**) *G. massiliensis* Marseille-Q2328^T^ and (**d**) *G. phocaeensis* Marseille-Q9179^T^ generated by the CGView server.

The genome length of strain Marseille-Q9181^T^ is 1,702,271 bp, assembled into 12 contigs, with a G+C content of 41.4 mol%. The gene prediction analysis of strain Marseille-Q9181^T^ reports 1,374 predicted genes, including 1,323 protein-coding genes, 51 RNA genes (5 rRNA, 45 tRNA and 1 tmRNA) and 7 pseudogenes (Table 5 and Fig. 2a). Strain Marseille-QA0894^T^ has a genome length of 1,505,165 bp, assembled into 12 contigs, with a G+C content of 43.2 mol%. Strain Marseille-QA0894^T^ comprises 1,138 predicted genes, including 1,207 protein-coding genes, 49 RNA genes (3 rRNA, 45 tRNA and 1 tmRNA) and 5 pseudogenes (Table 5 and Fig. 2b). The genome length of strain Marseille-Q2328^T^ is 1,720,610 bp, assembled into 32 contigs, with a G+C content of 45.3 mol%. Strain Marseille-Q2328^T^ has 1,380 predicted genes, with 1,331 protein-coding genes, 49 RNA sequences (3 rRNA, 45 tRNA and 1 tmRNA), 7 pseudogenes and 1 repeat region (Table 5 and Fig. 2c). Strain Marseille-Q9179^T^ has a genome length of 1,565,522 bp, assembled into 12 contigs, with a G+C content of 42.3 mol%. Strain Marseille-Q9179^T^ has 1,227 predicted genes, including 1,178 protein-coding genes, 49 RNA sequences (3 rRNA, 45 tRNA and 1 mRNA), 4 pseudogenes and 1 repeat region (Table 5 and Fig. 2d).

**Table 5.** Genome properties of strains *G. lacydonensis* Marseille-Q9181^T^, *G. bretellae* Marseille-QA0894^T^, *G. massiliensis* Marseille-Q2328^T^ and *G. phocaeensis* Marseille-Q9179^T^ compared to other *Gardnerella* species officially described (*G. vaginalis* ATCC 14018^T^, *G. leopoldii* UGent 06.41^T^, *G. swidsinskii* GS 9838-1^T^, *G. piotii* 18.01^T^, *G. greenwoodii* c31Ua_26^T^ and *G. pickettii* c17Ua_112^T^)

Genome features	*G. lac* Marseille-Q9181^T^	*G. bret* Marseille-QA0894^T^	*G. mas* Marseille-Q2328^T^	***G. pho*** **Marseille-Q9179^T^**	*G. vag* ATCC 14018**^T^**	*G. leo* UGent 06.41**^T^**	*G. swid* GS 9838–1**^T^**	*G. piot* UGent 18.01**^T^**	*G. green* c31Ua_26**^T^**	*G. pick* c17Ua_112**^T^**
**Genome size (bp)**	1,702,271	1,505,165	1,720,610	1,565,522	1,667,406	1,563,545	1,622,089	1,514,270	1,511,834	1,509,345
**Contigs**	12	12	32	12	1	1	9	5	11	3
**G+C content (mol%)**	41.4	43.2	45.3	42.3	41.4	42.1	41.9	42.5	43.3	42.5
**Genes**	1,374	1,186	1,380	1,227	1,320	1,239	1,327	1,210	1,176	1,204
**CDSs**	1,323	1,138	1,331	1,178	1,268	1,187	1,277	1,159	1,127	1,155
**RNAs**	51	48	49	49	52	52	50	51	49	49
**rRNAs (5S, 16S, 23S)**	5	3	3	3	6	6	4	5	3	3
**tRNAs**	45	44	45	45	45	45	45	45	45	45
**tmRNA**	1	1	1	1	1	1	1	1	1	1
**Repeat region**	0	0	1	1	1	1	2	1	2	0
**N50 length**	439,839	304,156	166,989	425,876	1,667,384	948,424	432,172	1,406,824	472,464	1,398,357
**L50**	2	3	4	2	1	1	2	1	2	1
**Accession number**	JBKQXT000000000	JBKQXU000000000	CALNWW000000000	JBKQXS000000000	QJUZ00000000	CP029984	QJVB00000000	QJUV00000000	JAKNCL000000000	JAKNCU000000000

N50: sequence length of the shortest contig at 50% of the total length of the assembly. L50: number of the smallest number of contigs whose sum of lengths is half the size of the genome.

### Phylogenetic tree

**Fig. 3.**
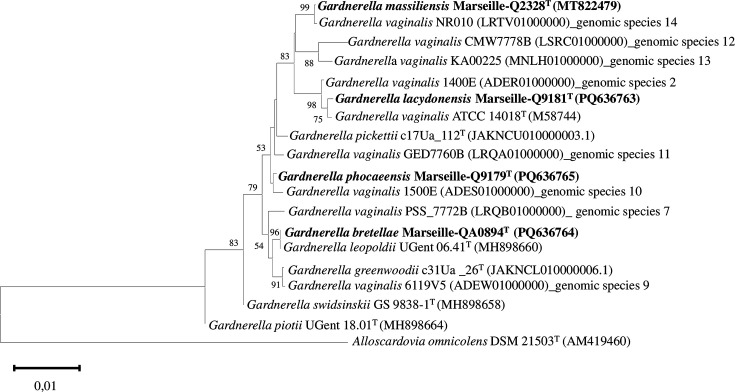
Phylogenetic tree based on 16S rRNA gene sequences showing the evolutionary relationships of *G. lacydonensis* Marseille-Q9181^T^, *G. bretellae* Marseille-QA0894^T^, *G. massiliensis* Marseille-Q2328^T^ and *G. phocaeensis* Marseille-Q9179^T^ with officially described and closely related *Gardnerella* strains. The tree was deduced using the neighbour-joining method. Evolutionary distances were calculated using the Tamura 3-parameter method. Bootstrap percentages (based on 1,000 replications) are indicated at the nodes.

The phylogenetic 16S rRNA tree illustrated the relationship between strains Marseille-Q9181^T^, Marseille-QA0894^T^, Marseille-Q2328^T^ and Marseille-Q9179^T^ and closely related species within the genus *Gardnerella* (Fig. 3). Comparative analysis of 16S rRNA sequences using the blast tool revealed that strains Marseille-Q9181^T^, Marseille-QA0984^T^, Marseille-Q2328^T^ and Marseille-Q9179^T^ showed sequence homologies of 99.78%, 100%, 100% and 99.80% with strains *G. vaginalis* ATCC 14018^T^, *G. leopoldii* UGent 06.41^T^, *G. vaginalis* NR010 and *G. vaginalis* 1500E, respectively.

### Functional annotation

**Fig. 4.**
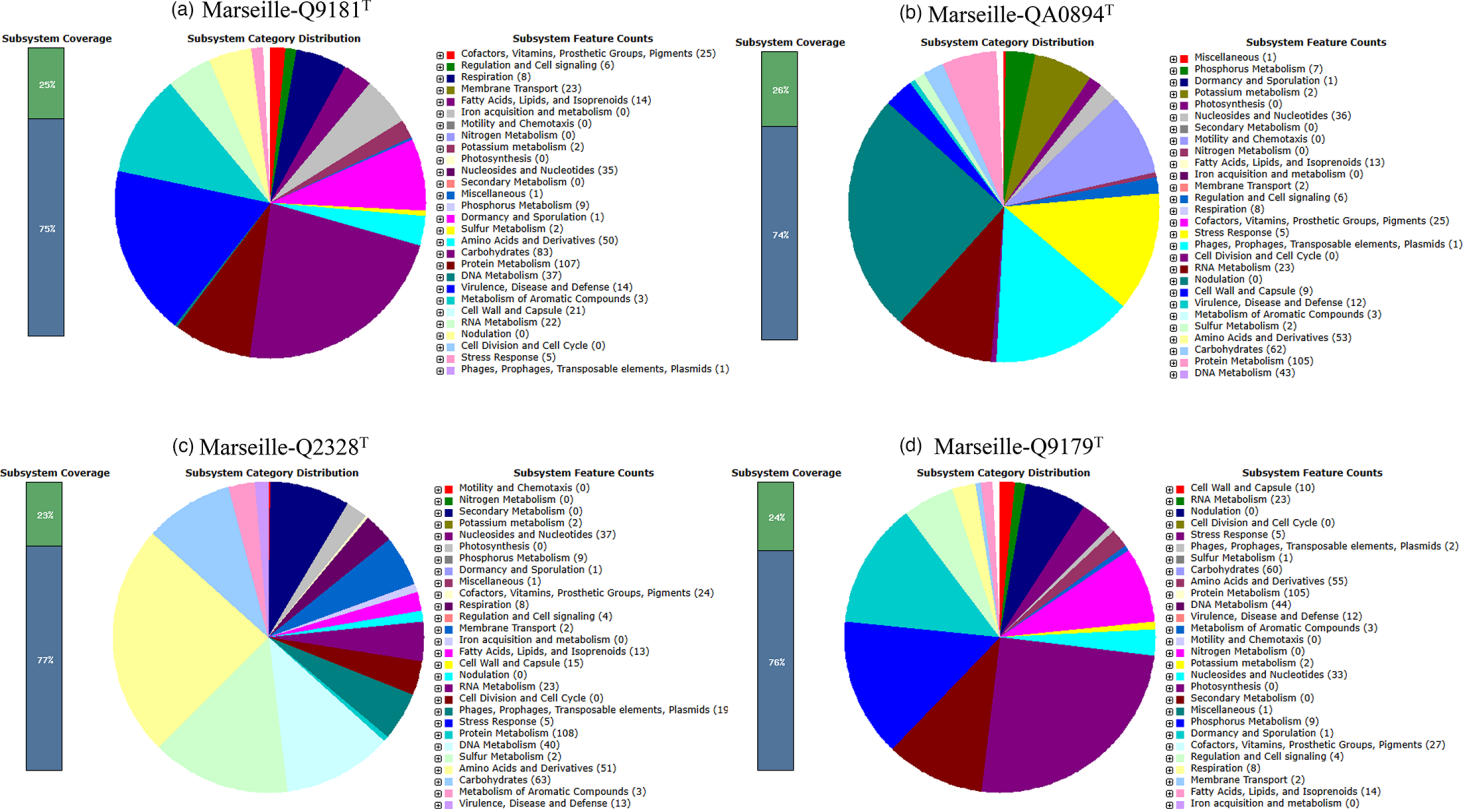
Genome subsystem coverage, distribution of subsystem categories and number of subsystem features of strains (**a**) *G. lacydonensis* Marseille-Q9181^T^, (**b**) *G. bretellae* Marseille-QA0894^T^, (**c**) *G. massiliensis* Marseille-Q2328^T^ and (**d**) *G. phocaeensis* Marseille-Q9179^T^ by RAST.

The characteristics of the protein-coding gene subsystems detected by RAST in the four species studied were mainly related to subsystems involved in the metabolism of proteins, carbohydrates, amino acids and their derivatives, DNA, nucleosides and nucleotides metabolism, membrane transport, cell wall, capsule, cofactors, vitamins, prosthetic group, pigments and RNA metabolism. However, there were fewer genes encoding cell wall and capsule in strains Marseille-Q2328^T^ and Marseille-Q9179^T^. Genes encoding membrane transport were less prevalent in strains Marseille-QA0894^T^ and Marseille-Q9179^T^. In all four strains, genes encoding secondary metabolism, iron acquisition and metabolism, cell division, the cell cycle, photosynthesis, motility and chemotaxis and nitrogen metabolism were absent. The distribution of subsystem categories and the number of subsystem features for each bacterial strain are shown in Fig. 4. The distribution of COGs, shown in Table 6, is similar for all four strains. The number of genes encoding carbohydrate transport and metabolism was more predominant in strain Marseille-Q9181^T^. The number of genes encoding for mobilome was prevalent to Marseille-QA0894^T^ and Marseille-Q2328^T^. A high number of COG functional genes involved in replication, recombination and repair were found in Marseille-Q2328^T^. No genes encoding chromatin structure and dynamics or nuclear structure were found in any of the four strains.

**Table 6.** Number of genes associated with the 25 general COG functional categories for strains *G. lacydonensis* Marseille-Q9181^T^, *G. bretellae* Marseille-QA0894^T^, *G. massiliensis* Marseille-Q2328^T^ and *G. phocaeensis* Marseille-Q9179^T^

Code	COG function	*G. lacydonensis* Marseille-Q9181^T^	*G. bretellae* Marseille-QA0894^T^	*G. massiliensis* Marseille-Q2328^T^	*G. phocaeensis* Marseille-Q9179^T^
[A]	RNA processing and modification	1	1	1	1
[B]	Chromatin structure and dynamics	0	0	0	0
[C]	Energy production and conversion	24	23	23	23
[D]	Cell cycle control, cell division, chromosome partitioning	15	15	16	14
[E]	Amino acid transport and metabolism	62	65	64	65
[F]	Nucleotide transport and metabolism	49	50	52	47
[G]	Carbohydrate transport and metabolism	124	78	74	77
[H]	Coenzyme transport and metabolism	40	35	43	42
[I]	Lipid transport and metabolism	28	25	25	27
[J]	Translation, ribosomal structure and biogenesis	144	144	142	142
[K]	Transcription	49	39	42	39
[L]	DNA replication, recombination and repair	64	63	80	61
[M]	Cell wall/membrane/envelope biogenesis	76	72	72	69
[N]	Cell motility	3	2	2	2
[O]	Posttranslational modification, protein turnover, chaperones	51	49	53	46
[P]	Inorganic ion transport and metabolism	40	37	37	39
[Q]	Secondary metabolite biosynthesis, transport and catabolism	2	1	1	2
[R]	General function prediction only	43	37	37	39
[S]	Function unknown	24	24	24	21
[T]	Signal transduction mechanisms	28	27	27	27
[U]	Intracellular trafficking, secretion and vesicular transport	8	8	8	8
[V]	Defence mechanisms	47	43	39	44
[X]	Mobilome: prophages, transposons	9	18	29	7
[Y]	Nuclear structure	0	0	0	0
[Z]	Cytoskeleton	0	0	0	0
–	Not in COGs	128	119	126	113

### Genome comparison of the four strains

**Fig. 5.**
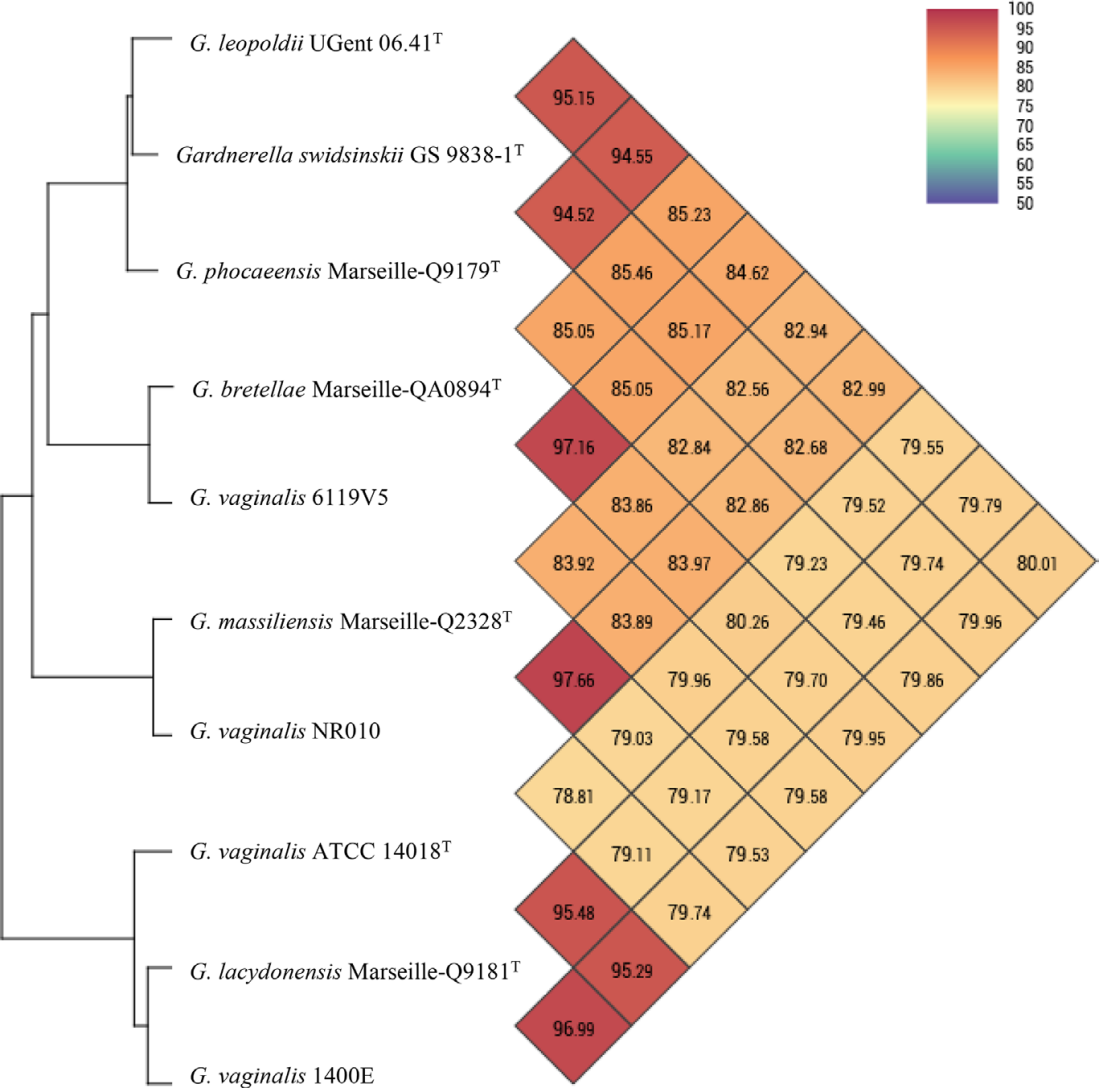
Heat map showing the average nucleotide identity based on calculated orthology (OrthoANI) of strains *G. lacydonensis* Marseille-Q9181^T^, *G. bretellae* Marseille-QA0894^T^, *G. massiliensis* Marseille-Q2328^T^ and *G. phocaeensis* Marseille-Q9179^T^ relative to the closest *Gardnerella* species.

The genomic characteristics of the 4 strains were compared with the genomes of the 14 *Gardnerella* genomic species available on NCBI (Table S1). dDDH and OrthoANI values are summarized in Table 7. OrthoANI data are also reported in a heat map (Fig. 5). A dDDH value of 72.1% was observed between strain Marseille-Q9181^T^ and strain *G. vaginalis* 1400E (genomic species 2). Furthermore, an OrthoANI value (96.99%) was observed for strain Marseille-Q9181^T^ and strain *G. vaginalis* 1400E (genomic species 2). A dDDH value of 72.4% was observed between strain Marseille-QA0894^T^ and strain *G. vaginalis* 6119V5 (genomic species 9). Furthermore, the highest OrthoANI value (97.16%) was observed for strain Marseille-QA0894^T^ and strain *G. vaginalis* 6119V5 (genomic species 9). A dDDH value of 75.5% was observed between strain Marseille-Q2328^T^ and strain *G. vaginalis* NR010 (genomic species 14). Furthermore, the highest OrthoANI value (97.66%) was observed for strain Marseille-Q2328^T^ and strain *G. vaginalis* NR010 (genomic species 14). The highest dDDH values for strain Marseille-Q9179^T^ were 57.6% with *G. swidsinskii* GS 9838-1^T^ and 57.3% with *G. leopoldii* UGent 06.41^T^. OrthoANI values below 95% were observed between strain Marseille-Q9179^T^ and *G. swidsinskii* GS 9838-1^T^, as well as strain *G. leopoldii* UGent 06.4^T^.

**Table 7.** dDDH values (%) and OrthoANI between the strains studied and 14 *Gardnerella* genomic species

Query strains	*Gardnerella* subject strains	dDDH (%)	OrthoANI (%)	Difference G+C%
***G. lacydonensis* Marseille-Q9181^T^**	*G. vaginalis* ATCC 14018^T^	61.5	95.48	0.04
	*G. vaginalis* 1400E (genomic species 2)	72.1	96.99	0.22
	*G. pickettii* c17Ua_112^T^	32.1	87.18	1.08
	*G. piotii* UGent 18.01^T^	32.2	86.85	1.07
	*G. leopoldii* UGent 06.41^T^	23	79.79	0.7
	*G. swidsinskii* GS 9838-1^T^	26.8	79.74	0.44
	*G. vaginalis* PSS_7772B (genomic species 7)	27.6	79.68	1.83
	*G. greenwoodii* c31Ua_26^T^	26.3	79.79	1.9
	*G. vaginalis* 6119V5 (genomic species 9)	26	79.58	1.86
	*G. vaginalis* 1500E (genomic species 10)	27	79.64	1.56
	*G. vaginalis* GED7760B (genomic species 11)	30.2	85.89	1.87
	*G. vaginalis* CMW7778B (genomic species 12)	27.3	78.15	3.39
	*G. vaginalis* KA00225 (genomic species 13)	28.7	79.73	0.59
	*G. vaginalis* NR010 (genomic species 14)	26.5	79.11	4.04
	*G. bretellae* Marseille-QA0894^T^	26.5	79.7	1.79
	*G. massiliensis* Marseille-Q2328^T^	26.7	79.17	3.85
	*G. phocaeensis* Marseille-Q9179^T^	27.1	79.46	0.85
***G. bretellae* Marseille-QA0894^T^**	*G. vaginalis* ATCC 14018^T^	27.5	80.26	1.83
	*G. vaginalis* 1400E (genomic species 2)	27	79.95	2.01
	*G. pickettii* c17Ua_112^T^	26.7	79.42	0.71
	*G. piotii* UGent 18.01^T^	26.7	79.62	0.75
	*G. leopoldii* UGent 06.41^T^	28.2	85.23	1.1
	*G. swidsinskii* GS 9838-1^T^	29.5	85.46	1.35
	*G. vaginalis* PSS_7772B (genomic species 7)	30.8	85.22	0.26
	*G. greenwoodii* c31Ua_26^T^	58.9	95.02	0.11
	*G. vaginalis* 6119V5 (genomic species 9)	72.4	97.16	0.07
	*G. vaginalis* 1500E (genomic species 10)	60.2	95.31	0.24
	*G. vaginalis* GED7760B (genomic species 11)	26.9	79.68	0.08
	*G. vaginalis* CMW7778B (genomic species 12)	26.2	78.45	5.18
	*G. vaginalis* KA00225 (genomic species 13)	28.4	81.12	2.38
	*G. vaginalis* NR010 (genomic species 14)	29	83.97	2.24
	*G. massiliensis* Marseille-Q2328^T^	29.3	83.92	2.06
	*G. phocaeensis* Marseille-Q9179^T^	29	85.05	0.94
***G. massiliensis* Marseille-Q2328^T^**	*G. vaginalis* ATCC 14018^T^	26.8	79.03	0.58
	*G. vaginalis* 1400E (genomic species 2)	28.1	79.53	4.07
	*G. pickettii* c17Ua_112^T^	26.5	78.79	2.77
	*G. piotii* UGent 18.01^T^	26.7	78.8	2.81
	*G. leopoldii* UGent 06.41^T^	28.1	82.94	3.16
	*G. swidsinskii* GS 9838-1^T^	27.2	82.56	3.41
	*G. vaginalis* PSS_7772B (genomic species 7)	27.7	83.16	2.32
	*G. greenwoodii* c31Ua_26^T^	29.1	83.65	1.95
	*G. vaginalis* 6119V5 (genomic species 9)	29.4	83.92	1.99
	*G. vaginalis* 1500E (genomic species 10)	28.4	83.37	2.3
	*G. vaginalis* GED7760B (genomic species 11)	26.5	78.91	1.98
	*G. vaginalis* CMW7778B (genomic species 12)	27.7	77.91	7.28
	*G. vaginalis* KA00225 (genomic species 13)	30.1	80.5	4.45
	*G. vaginalis* NR010 (genomic species 14)	75.5	97.66	0.18
	*G. phocaeensis* Marseille-Q9179^T^	27.4	82.84	3
***G. phocaeensis* Marseille-Q9179^T^**	*G. vaginalis* ATCC 14018^T^	26.6	79.23	1.9
	*G. vaginalis* 1400E (genomic species 2)	26.9	79.86	1.07
	*G. pickettii* c17Ua_112^T^	26.3	79.09	0.23
	*G. piotii* UGent 18.01^T^	26.2	78.93	0.18
	*G. leopoldii* UGent 06.41^T^	57.3	94.55	0.16
	*G. swidsinskii* GS 9838-1^T^	57.6	94.52	0.42
	*G. vaginalis* PSS_7772B (genomic species 7)	28.5	84.01	0.68
	*G. greenwoodii* c31Ua_26^T^	28.9	85.09	1.05
	*G. vaginalis* 6119V5 (genomic species 9)	28.6	85.05	1.01
	*G. vaginalis* 1500E (genomic species 10)	28.7	84.9	0.7
	*G. vaginalis* GED7760B (genomic species 11)	26.1	78.89	1.02
	*G. vaginalis* CMW7778B (genomic species 12)	26	78.42	4.25
	*G. vaginalis* KA00225 (genomic species 13)	31.1	82.35	1.45
	*G. vaginalis* NR010 (genomic species 14)	27.2	82.86	3.18

Only the strain Marseille QA0894^T^ had one CRISPR sequence and one CRISPR-Cas type IE. The polyketosynthase-related Type III gene cluster was predicted in Marseille-Q9181^T^. Bacteriocin, plasmid, phage and insert sequence were not identified. The gene coding for *nanH1* was present in Marseille-Q9181^T^, Marseille-QA0894^T^ and Marseille-Q2328^T^. No gene coding for sialidase was found in Marseille-Q9179^T^. Vaginolysin type 1B was found in Marseille-Q9181^T^ and Marseille-QA0894^T^. Vaginolysin type 1A was found in Marseille-Q2328^T^. Marseille-Q9179^T^ was aligned with vaginolysin type 2 possessing glutamic acid at position −1 and a substitution of valine by alanine at position 6 in the undecapeptide region (Fig. S2). Structural modelling of the candidate proteins vaginolysin and sialidase using PHYRE 2.2 enabled functional confirmation, with a confidence level of 100%.

## Discussion

*G. vaginalis* species is categorized in the phylum *Actinomycetota*, class *Actinomycetes*, order *Bifidobacteriales*, family *Bifidobacteriaceae* and genus *Gardnerella* [46]. Until recently, the genus *Gardnerella* comprised 14 genomic species, 6 of which have got subsequently validly published names [10,11]. Gardner and Dukes were the first to demonstrate the presence of *G. vaginalis* in women with bacterial vaginosis, formerly known as non-specific bacterial vaginitis [47]. Later, several studies showed a significant association between *G. vaginalis* and bacterial vaginosis [2,48, 49]. Bacterial vaginosis is a dysbiosis characterized by a decrease in lactobacilli and the proliferation of several anaerobic bacteria, such as *G. vaginalis*, *Fannyhessea vaginae*, *Prevotella bivia*, *Mobiluncus curtisii* and many others [50,52].

Recent studies revealed that the complex of strains previously attributed to a single species, *G. vaginalis*, and associated with bacterial vaginosis could represent multiple similar but distinct and sometimes neighbour species [4,7, 10, 53]. Several genetically distinct groups have already been described as new species (*G. picketti*, formerly genomic species 3, *G. piotii*, formerly genomic species 4, * G. leopoldii*, formerly genomic species 5, *G. swidsinskii*, formerly genomic species 6, and *G. greenwoodii*, formerly genomic species 8). Other genomic species are not yet officially described. Here, we continue to dissect former genomic species of *G. vaginalis* that may represent different species according to modern criteria. We analysed four *Gardnerella* strains isolated from vagina and blood in Marseille, France, and characterized them.

Strains Marseille-Q9181^T^, Marseille-QA0894^T^ and Marseille-Q9179^T^ have a whitish to greyish morphology almost identical to that of *G. vaginalis*, *G. piotii*, *G. leopoldii* and *G. swidsinskii* as described by Vaneechoutte *et al*. [10], which limits the potential for identifying *Gardnerella* species based solely on the appearance of colonies isolated in culture. Previous studies have used β-galactosidase as a biochemical test to differentiate *Gardnerella* species [3,10, 11]. Our results showed that strains Marseille-QA0894^T^, Marseille-Q2328^T^ and Marseille-Q9179^T^ were negative for β-galactosidase, while strain Marseille-Q9181^T^ exhibited positive β-galactosidase activity. These data support the conclusion that the β-galactosidase test is no longer suitable for identifying *Gardnerella* species. The strains Marseille-Q9181^T^, Marseille-QA0894^T^, Marseille-Q2328^T^ and Marseille-Q9179^T^ appear as Gram-negative coccobacilli. They can grow at 5% CO_2_ and show no activity for catalase, oxidase and β-glucosidase. These phenotypic characteristics are similar to those observed in other species of the genus *Gardnerella* [10,47, 54].

A recent study updated the MALDI Biotyper database to distinguish between the *G. vaginalis*/*G. piotii* and *G. leopoldii*/*G. swidsinskii* pairs for routine diagnosis [55]. However, our results showed that even with an improved MALDI Biotyper database, it was not possible to accurately distinguish *Gardnerella* species.

Phylogenetic analysis based on the comparison of the 16S rRNA gene revealed that strains Marseille-Q9181^T^, Marseille QA0894^T^, Marseille-Q2328^T^ and Marseille-Q9179^T^ were more than 98.5% similar to their closest phylogenetic neighbour. However, this criterion is insufficient to establish a new species in *Gardnerella* as mentioned in previous studies [10,14, 56]. According to the generally accepted criteria for bacterial species delimitation, namely dDDH 70% and ANI 96% [34,57], strains Marseille-Q9181^T^, Marseille-QA0894^T^ and Marseille-Q2328^T^ belong to *Gardnerella* genomic species 2, 9 and 14, respectively (dDDH ≥70% and ANI ≥96%). However, the strain Marseille-Q9179^T^ exhibits dDDH and ANI values below the thresholds established for species delimitation, confirming that this strain is a new species of the genus *Gardnerella*. Based on phenotypic, phylogenetic and genomic analyses, we describe four species of the genus *Gardnerella*: *G. lacydonensis* sp. nov., strain Marseille-Q9181^T^, belongs to the previously reported *Gardnerella* genomic species 2; *G. bretellae* sp. nov., strain Marseille-QA0894^T^, belongs to the previously reported *Gardnerella* genomic species 9 [10]; *G. massiliensis* sp. nov., strain Marseille-Q2328^T^, belongs to the previously reported *Gardnerella* genomic species 14 [14]; and *G. phocaeensis* sp. nov., strain Marseille-Q9179^T^, is a new species of the genus *Gardnerella* that may also be represented as *Gardnerella* genomic species 15.

The genome analyses show that the investigated strains have a genome size of 1.491 to 1.716 Mb and a G+C content from 41.1 mol% to 43.4 mol% that is generally comparable to the values described for the genus *Gardnerella* [58]. However, the strain Marseille-Q2328^T^ is distinguished by a significantly higher G+C content (45.3 mol%) than the officially described strains [10,11]. In the genus *Gardnerella*, variation in G+C content has already been correlated with isolated genomic clades specifically adapted to different ecological niches, suggesting a role in adaptive divergence [58]. It should be noted that the strain with the highest G+C content (Marseille-Q2328^T^) was isolated from blood, in contrast to the others, which were isolated from the vagina. Furthermore, evidence of mobile subsystems and a high number of genes associated with DNA replication, recombination and repair in the strains Marseille-QA0894^T^ and Marseille-Q2328^T^ highlights a diverse and highly adaptable pangenome within these strains, characterized by frequent horizontal gene acquisition, genetic mobility and genome expansion [58]*.*

Sialidase has harmful effects on the vaginal mucosa, facilitating the adhesion of bacteria to the vaginal epithelium and the formation of biofilms [59,60]. Previously, three sialidase enzymes (*nanH1*, *nanH2* and *nanH3*) have been identified in strains of the genus *Gardnerella* [43]. The *nanH1* gene, encoding sialidase A, was detected; however, none of the strains possessed a gene encoding the active enzyme (*nanH2* and *nanH3*) [43]. Thus, these results show that the four strains of *Gardnerella* do not possess sialidase activity. The four strains also possess the vaginolysin gene, as observed in other *Gardnerella* species [42]. The species of the genus *Gardnerella* produces vaginolysin, a toxin that is a cholesterol-dependent cytolysin [61]. According to a recent study, *Gardnerella* species have five different forms of vaginolysin: type 1A, type 1B, type 1C, type 2 and type 3 [42]. However, the strain Marseille-Q9179^T^ exhibits unique variability in the type 2 undecapeptide region of vaginolysin, which could indicate the existence of a new subclass of vaginolysin.

### Description of *Gardnerella lacydonensis* sp. nov. (formerly known as *Gardnerella* genomic species 2)

*Gardnerella lacydonensis* (la.cy.don.en’sis. sis, N.L. fem. adj. *lacydonensis*, from *Lacydon*, the name of the ancient port of Marseille, the French city where the strain was first described).

Cells are facultative anaerobic and microaerophilic, non-motile, non-spore-forming and Gram-negative coccobacilli. The cells are 1.39±0.4 µm long and 0.86±0.07 µm wide. Catalase and oxidase activities are negative. Colonies are round, greyish to whitish and opaque on PolyViteX chocolate agar, Columbia CNA agar supplemented with 5% sheep blood and Columbia agar enriched with 5% sheep blood after 48 h of incubation. Growth occurs in a temperature range of 28–37 °C (optimum 37 °C), at a pH of 7.5 to 8.5 (optimum 7.5) and 0.5% NaCl. Using API strips, positive reactions are observed for d-glucose, d-mannitol, lactose, d-sucrose, d-maltose, salicin, aesculin, iron citrate, glycerol, d-cellobiose, d-mannose, d-melezitose, d-sorbitol, starch, alkaline phosphatase, leucine arylamidase, valine arylamidase, acid phosphatase, naphthol-AS-BI-phosphohydrolase, α-glucosidase, ß-galactosidase and d-mannosidase. The predominant fatty acids are C_16 : 0_, C_18 : 1_ ω9c and C_18 : 0_. The genome size is 1.7 Mbp, with a G+C content of 41.4 mol%. The type strain Marseille-Q9181^T^ (=CSUR Q9181^T^=CECT 31121^T^) was isolated from a vaginal sample. The 16S rRNA and genome sequences were deposited in GenBank under accession numbers PQ636763 and JBKQXT000000000, respectively.

### Description of *Gardnerella bretellae* sp. nov. (formerly known as *Gardnerella* genomic species 9)

*Gardnerella bretellae* (bre.tel’lae, N.L. gen. n. *bretellae*, honouring Florence Bretelle for her contribution to the description of vaginal flora).

Cells are strictly anaerobic and microaerophilic, non-motile, non-spore-forming and Gram-negative coccobacilli. Cells are 0.73±0.13 µm long and 0.5±0.06 µm wide. Catalase and oxidase activities are negative. Growth is observed at 37–42 °C (optimum 37 °C), pH 6.5–8.5 (optimum 7.5) and 0.5% NaCl. Bacterial colonies are round, greyish to whitish and opaque on PolyViteX chocolate agar, Columbia CNA agar supplemented with 5% sheep blood and Columbia agar enriched with 5% sheep blood after 48 h of incubation at 37 °C. Using API strips, positive reactions are observed for d-glucose, d-mannitol, lactose, d-sucrose, d-maltose, salicin, aesculin, iron citrate, glycerol, d-cellobiose, d-mannose, d-melezitose and d-sorbitol, starch, alkaline phosphatase, leucine arylamidase, valine arylamidase, acid phosphatase, naphthol-AS-BI-phosphohydrolase and α-glucosidase. The predominant fatty acids are C_16 : 0_, C_18 : 1_ ω9c and C_18 : 0_. The genome size is 1.5 Mbp, with a G+C content of 43.2 mol%. The type strain Marseille-QA0894^T^ (=CSUR QA0894^T^=CECT 31122^T^) was isolated from a vaginal sample. The 16S rRNA and genome sequences were deposited in GenBank under accession numbers PQ636764 and JBKQXU000000000, respectively.

### Description of *Gardnerella massiliensis* sp. nov. (formerly known as *Gardnerella* genomic species 14)

*Gardnerella massiliensis* (mas.si.li.en’sis. N.L. fem. adj. *massiliensis*, from *Massilia*, the Latin name of Marseille in France, where the bacterium was first described).

Cells are facultative anaerobic and microaerophilic, non-motile, non-spore-forming and Gram-negative coccobacilli. Cells are 3±0.64 µm long and 1.5±0.09 µm wide. Catalase and oxidase activities are negative. Growth occurs in a temperature range of 28–42 °C (optimum 37 °C) at a pH of 6.5 to 8.5 (optimum 7.5) and 0.5% NaCl. Bacterial colonies are small, translucent, almost transparent and circular on Columbia agar enriched with 5% sheep’s blood and Columbia CNA agar supplemented with 5% sheep’s blood, after 48 h of incubation at 37 °C. Using API strips, positive reactions are observed for d-glucose, d-mannitol, lactose, d-sucrose, d-maltose, salicin, aesculin, iron citrate, glycerol, d-cellobiose, d-mannose, d-melezitose, d-sorbitol, starch, alkaline phosphatase, leucine arylamidase, valine arylamidase, acid phosphatase, naphthol-AS-BI-phosphohydrolase and α-glucosidase. The most abundant fatty acids are C_16 : 0_, C_18 : 1_ ω9c and C_18 : 0_. The genome size is 1.7 Mbp, with a G+C content of 45.3 mol%. The type strain Marseille-Q2328^T^ (=CSUR Q2328^T^=CECT 30239^T^) was isolated from blood. The 16S rRNA and genome sequences were deposited in GenBank under accession numbers MT822479 and CALNWW000000000, respectively.

### Description of *Gardnerella phocaeensis* sp. nov.

*Gardnerella phocaeensis* (pho.cae.en’sis, N.L. fem. adj. *phocaeensis*, referring to Phocaea, the name of the Ionian Greek city where the founders of Marseille came from. The strain was isolated in Marseille).

Cells are facultative anaerobic and microaerophilic, non-motile, non-spore-forming and Gram-negative coccobacilli. Cells are 1±0.05 µm long and 0.54±0.04 µm wide. Catalase and oxidase activities are negative. Colonies are greyish to whitish in appearance and circular on Columbia agar enriched with 5% sheep’s blood, PolyViteX chocolate agar and Columbia CNA agar enriched with 5% sheep’s blood, after 48 h of incubation at 37 °C.

Growth occurs in a temperature range of 28–42 °C (optimum 37 °C), at a pH of 7.5 to 8.5 (optimum 7.5) and 0.5% NaCl. Using API strips, positive reactions are observed for d-glucose, d-mannitol, lactose, d-sucrose, d-maltose, salicin, aesculin, iron citrate, glycerol, d-cellobiose, d-mannose, d-melezitose, d-sorbitol, starch, l-rhamnose, d-trehalose, alkaline phosphatase, leucine arylamidase, valine arylamidase, acid phosphatase, naphthol-AS-BI-phosphohydrolase, α-glucosidase, esterase (C4), esterase-lipase (C8), lipase (C14) and ß-glucuronidase. The most abundant fatty acids are C_16 : 0_, C_18 : 1_ ω9c, C_18 : 0_ and C_14 : 0_. The genome size is 1.5 Mbp, with a G+C content of 42.3 mol%. The type strain Marseille-Q9179^T^ (=CSUR Q9179^T^=CECT 31120^T^) was isolated from a vaginal sample. The 16S rRNA and genome sequences were deposited in GenBank under accession numbers PQ636765 and JBKQXS000000000, respectively.

## Supplementary material

10.1099/ijsem.0.007028 Uncited Supplementary Material 1.

## Abbreviations

COGs: Clusters of Orthologous Genes
dDDH: digital DNA–DNA hybridization
RAST: Rapid Annotation Using Subsystem Technology
SEM: scanning electron microscopy
