# Supplementary material for: Further dissection of Gardnerella vaginalis: description of Gardnerella lacydonensis sp. nov. (formerly genomic species 2), Gardnerella bretellae sp. nov. (formerly genomic species 9), Gardnerella massiliensis sp. nov. (formerly genomic species 14) and Gardnerella phocaeensis sp. nov
Source: Int J Syst Evol Microbiol. 2026 Mar 4;76(3):007028. doi: 10.1099/ijsem.0.007028 (PMC12959845; doi:10.1099/ijsem.0.007028)

Supplementary Table 1. Genomes of the fourteen *Gardnerella* species used for genomic comparison (* represent genome of type strain).

Genomic species	*Gardnerella* species /strains	GenBank assembly	Size (Mb)	Number of contigs	G + C (%)	Protein-Coding Genes
**Genomic species 1**	*Gardnerella vaginalis* ATCC 14018^{T}*	GCA_001042655.1	1.7	1	41.5	1,260
**Genomic species 2**	*Gardnerella vaginalis* 1400E	GCA_000263495.1	1.7	28	41	1,337
**Genomic species 3**	*Gardnerella pickettii* c17Ua_112^{T}*	GCA_029226345.1	1.5	3	42.5	1,158
**Genomic species 4**	*Gardnerella piotii* UGent 18.01^{T}*	GCA_003397585.1	1.5	5	42.5	1,162
**Genomic species 5**	*Gardnerella leopoldii* UGent 06.41^{T}*	GCA_003293675.1	1.6	1	42	1,192
**Genomic species 6**	*Gardnerella swidsinskii* GS 9838-1^{T}*	GCA_003397705.1	1.6	9	42	1,281
**Genomic species 7**	*Gardnerella vaginalis* PSS_7772B	GCA_000414465.1	1.6	59	43	1,199
**Genomic species 8**	*Gardnerella greenwoodii* c31Ua_26^{T}*	GCA_029207615.1	1.5	11	43.3	1,132
**Genomic species 9**	*Gardnerella vaginalis* 6119V5	GCA_000263655.1	1.5	12	43.5	1,144
**Genomic species 10**	*Gardnerella vaginalis* 1500E	GCA_000263595.1	1.5	27	43	1,195
**Genomic species 11**	*Gardnerella vaginalis* GED7760B	GCA_001546455.1	1.5	60	43.5	1,150
**Genomic species 12**	*Gardnerella vaginalis* CMW7778B	GCA_001563665.1	1.6	61	38	1,185
**Genomic species 13**	*Gardnerella vaginalis* KA00225	GCA_002896555.1	1.7	52	41	1,265
**Genomic species 14**	*Gardnerella vaginalis* NR010	GCA_003408845.1	1.6	47	45.5	1,225

Supplementary Figure 1. Gram staining of strains *Gardnerella lacydonensis* Marseille-Q9181^{T}, *Gardnerella bretellae* Marseille-QA0894^{T}, *Gardnerella massiliensis* Marseille-Q2328^{T}, and *Gardnerella phocaeensis* Marseille-Q9179^{T} at 24 hours (1), 48 hours (2), and 72 hours (3).

A- *Gardnerella lacydonensis* Marseille-Q9181^{T}

B- *Gardnerella bretellae* Marseille-QA0894^{T}

C- *Gardnerella massiliensis* Marseille-Q2328^{T}

D- *Gardnerella phocaeensis* Marseille-Q9179^{T}

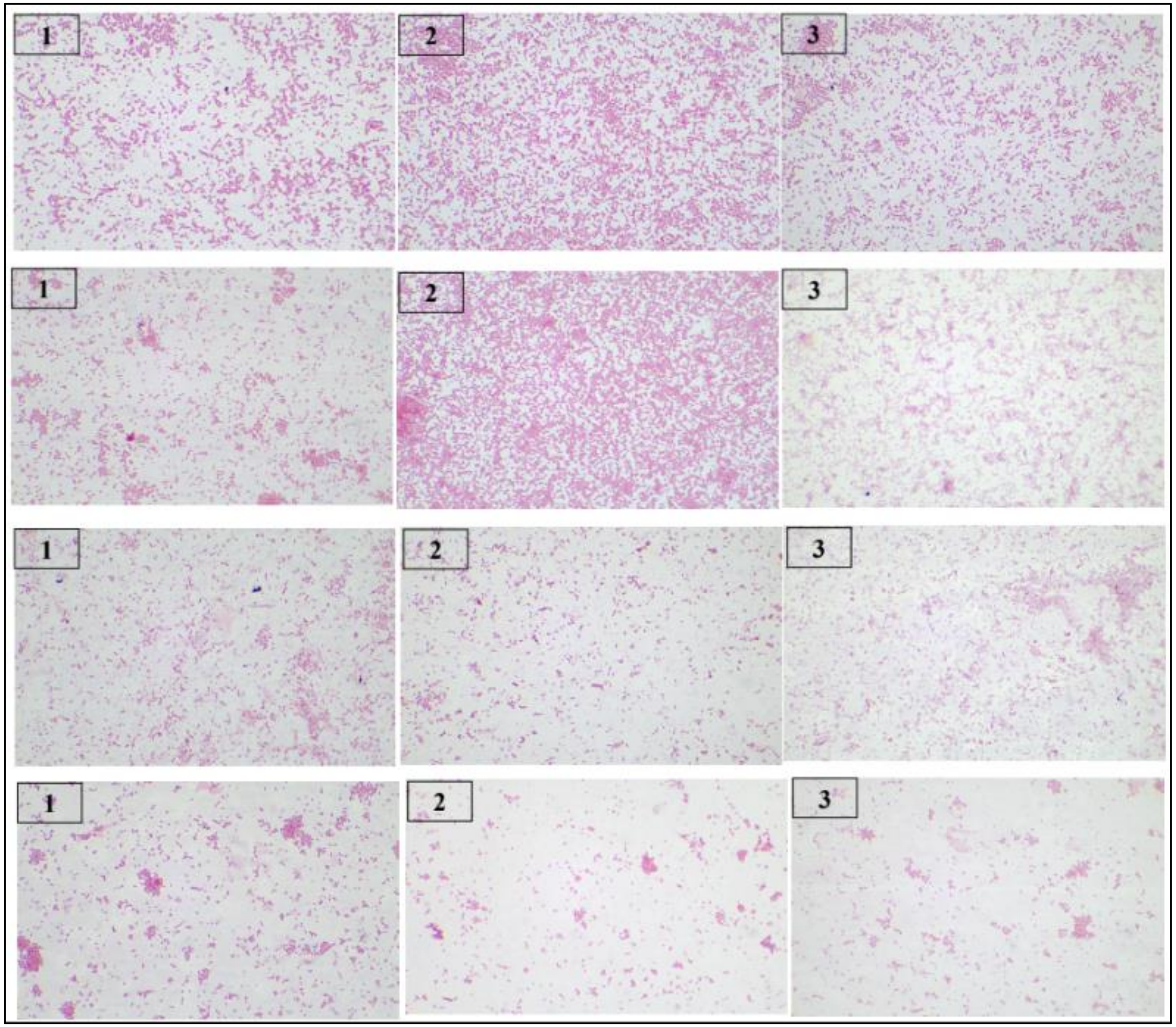

Supplementary Figure 2. Alignment of amino acid sequences of vaginolysin from strains *Gardnerella lacydonensis* Marseille-Q9181^{T}, *Gardnerella bretellae* Marseille-QA0894^{T}, *Gardnerella massiliensis* Marseille-Q2328^{T}, and *Gardnerella phocaeensis* Marseille-Q9179^{T} and other strains of species of the genus *Gardnerella*.

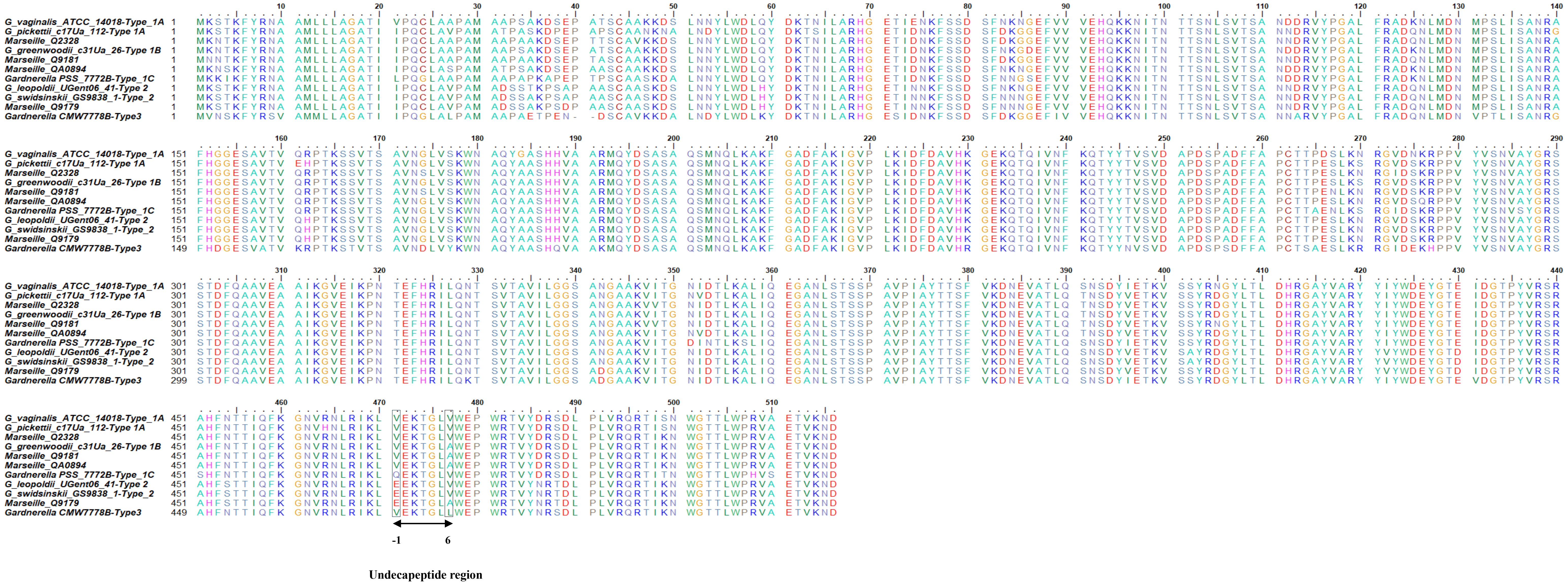

Supplement: Uncited Supplementary Material 1. [file ijsem-76-07028-s001.pdf]
